# Construction of a mortality risk prediction model for elderly people at risk of lobectomy for NSCLC

**DOI:** 10.3389/fsurg.2022.1055338

**Published:** 2023-01-06

**Authors:** Hongzhen Zhang, Dingfei Ren, Danqing Cheng, Wenping Wang, Yongtian Li, Yisong Wang, Dekun Lu, Feng Zhao

**Affiliations:** ^1^ Shanghai Fengxian District Central Hospital, Affiliated to Anhui University of Science and Technology, Fengxian, China; ^2^ Occupational Control Hospital of Huai He Energy Group, Huainan, China; ^3^ Graduate School of Bengbu Medical College, Bengbu, China; ^4^ Anhui University of Science and Technology College of Medicine, Huainan, China; ^5^ The First Hospital of Anhui University of Science & Technology (Huai nan First People's Hospital), Huainan, China

**Keywords:** SEER, NSCLC, lobectomy, propensity matching, columnar maps

## Abstract

**Background:**

An increasing number of lung cancer patients are opting for lobectomy for oncological treatment. However, due to the unique organismal condition of elderly patients, their short-term postoperative mortality is significantly higher than that of non-elderly patients. Therefore, there is a need to develop a personalised predictive tool to assess the risk of postoperative mortality in elderly patients.

**Methods:**

Information on the diagnosis and survival of 35,411 older patients with confirmed lobectomy NSCLC from 2009 to 2019 was screened from the SEER database. The surgical group was divided into a high-risk mortality population group (≤90 days) and a non-high-risk mortality population group using a 90-day criterion. Survival curves were plotted using the Kaplan-Meier method to compare the differences in overall survival (OS) and lung cancer-specific survival (LCSS) between the two groups. The data set was split into modelling and validation groups in a ratio of 7.5:2.5, and model risk predictors of postoperative death in elderly patients with NSCLC were screened using univariate and multifactorial logistic regression. Columnar plots were constructed for model visualisation, and the area under the subject operating characteristic curve (AUC), DCA decision curve and clinical impact curve were used to assess model predictiveness and clinical utility.

**Results:**

Multi-factor logistic regression results showed that sex, age, race, histology and grade were independent predictors of the risk of postoperative death in elderly patients with NSCLC. The above factors were imported into R software to construct a line graph model for predicting the risk of postoperative death in elderly patients with NSCLC. The AUCs of the modelling and validation groups were 0.711 and 0.713 respectively, indicating that the model performed well in terms of predictive performance. The DCA decision curve and clinical impact curve showed that the model had a high net clinical benefit and was of clinical application.

**Conclusion:**

The construction and validation of a predictive model for death within 90 days of lobectomy in elderly patients with lung cancer will help the clinic to identify high-risk groups and give timely intervention or adjust treatment decisions.

## Introduction

Lung cancer is one of the most common malignancies in the world, killing nearly 2 million people each year, mainly in the older age group of 65 years and above (1, 2). Non-small cell lung cancer (NSCLC) accounts for approximately 85% of lung cancers. With the accelerated ageing of the population and widespread screening by low-dose CT, the number of confirmed cases of NSCLC in the elderly has increased significantly (3, 4), posing a serious threat to human health and life.

Surgical resection is the treatment of choice for early-stage lung cancer, but there are many factors that affect post-operative survival due to the high mortality rate, multiple comorbidities and complex post-operative non-cancer related conditions that characterise the elderly lung cancer population (5–7). In 2021, Jiao et al. (8) constructed a prediction model combining radiological features and mortality risk parameters with a c-index as high as 0.734, which helped clinical identification of patients with early-stage NSCLC and was not an effective predictor of survival in NSCLC patients after surgery. A recent study (9) combined clinical and genomic features to construct a columnar graph model for risk stratification of early-stage NSCLC to assess the prognostic value of postoperative prognosis in NSCLC patients. These recent studies have used diverse approaches to construct postoperative predictive models for NSCLC, giving the models greater clinical predictive value, but these models are generally applicable to postoperative NSCLC patients without specific age differentiation.

Few current studies have investigated predictive models for monitoring risk factors for death and survival at 90 days after lobectomy in elderly (age ≥75 years) NSCLC. Based on the (SEER) database, this study retrospectively analysed the consultation and survival information of elderly patients with confirmed lobectomy NSCLC from 2009 to 2019 to develop a clinical line chart for predicting mortality after lobectomy in elderly NSCLC patients to facilitate clinical assessment of postoperative survival and development of individualised treatment strategies for elderly NSCLC patients.

## Materials and methods

### Data sources and study population

The data used in this study were all obtained from the National Cancer Institute's SEER database (SEER.cancer.gov) registry, a publicly available cancer database covering approximately 34.6% of the US population. Data from older patients with confirmed NSCLC from (2009 to 2019) were downloaded using SEER*Stat version 8.3.6 for analysis of the study. Screening inclusion criteria: (1) age ≥75 years; (2) meeting diagnostic criteria for NSCLC, confirmed clinically and pathologically; (3) Patients who underwent lobectomy for NSCLC; (4) Those with complete clinical data, including age, race, gender, tumour histological grade, molecular typing, postoperative survival time, and survival outcome. Exclusion criteria: (1) age <75 years; (2) previous history of lung tumors and other malignancies; (3) patients with non-NSCLC lobectomy; (4) those with incomplete clinical information. A total of 35,411 patients were eventually included as study subjects.

### PSM analysis

In this study, patients aged >75 years in the NSCLC surgery group were classified as non-high-risk for survival >90 days after surgery, and those with survival ≤90 days were classified as high-risk for death. To eliminate the influence of confounding factors on the study results, the 1:1 propensity score matching (PSM) was used to eliminate differences in baseline information between the two groups of data, and survival curves were plotted using the Kaplane-Meier method and log-rank tests were used to compare the differences in survival curves between the groups.

### Variable filtering

All enrolled CSCLC patients were further randomly split into a modelling and validation group in a ratio of 7.5:2.5. For the modelling group, univariate and multifactorial logistic regression were used to screen independent risk predictors for elderly patients undergoing lobectomy for NSCLC as the construct variables for the line graph model.

### Model construction and evaluation

Screened independent risk predictors for elderly patients undergoing lobectomy for NSCLC were incorporated into the prediction model, and the risk factors were presented visually using R software version 3.6.3 (http://www.r-project.org/). The predictive efficacy and clinical utility of the line graph prediction model was evaluated by analysing the receiver operating characteristic curve (ROC curve) and Decision Curve Analysis (DCA).

### Statistical analysis

All statistical analyses were performed using EmpowerStats (version 2.2) and R software (version 4.0.5). Count data were expressed as relative numbers and comparisons were made using the *χ*^2^ test; measurement data conforming to a normal distribution were expressed as (`x ± s) and measurement data not conforming to a normal distribution were expressed as M (P_25_, P_75_); one-way and multi-way logistic regression analyses were used to screen risk factors in CSCLC patients, and *P* < 0.05 was considered a statistically significant difference.

## Results

### Comparison of baseline information of elderly NSCLC cases

A total of 35,411 elderly patients with NSCLC aged ≥75 years in the SEER database were included in this study. To eliminate the influence of confounding factors on this study, we used a 1:1 propensity score matching (PSM) method for analysis. 21,658 patients in the NSCLC surgical group and 13,753 patients in the NSCLC non-surgical group before PSM were analyzed in both groups in terms of Age, Gender Marrital ststus, Race, Tuomor Position, Lung lobectomy, Histologic Type, Grade, AJCC-T, AJCC-N, AJCC-M were statistically significant (*P* < 0.05); after PSM, 9,058 patients in the NSCLC surgery group were compared with 9,058 patients in the NSCLC non-surgical group were successfully matched, with statistically significant differences in all variables except for the four confounding variables Gender, Involved lung lobes, AJCC-T, and AJCC-M, which were not statistically significant between groups (*P* > 0.05) (Table 1).

**Table 1:** Patient information based on their baseline features before and after 1:1 PSM in surgery and non surgery groups.

Variable	Beofre PSM		*P*-value	After PSM		*P*-value
	Surgery *n* = 21,658	Non-sugery *n* = 13,753		Surgery *n* = 9,058	Non-sugery *n* = 9,058	
Age	80.99 ± 4.44	79.32 ± 3.55	<0.001	81.41 ± 4.53	79.35 ± 3.56	<0.001
Gender (%)			<0.001			0.656
Female	10,202 (47.10%)	7,120 (51.77%)		4,563 (50.38%)	4,593 (50.71%)	
Male	11,456 (52.90%)	6,633 (48.23%)		4,495 (49.62%)	4,465 (49.29%)	
Marital Status			<0.001			<0.001
Married	10,156 (46.89%)	7,320 (53.22%)		4,340 (47.91%)	4,656 (51.40%)	
Signal	10,621 (49.04%)	5,855 (42.57%)		4,376 (48.31%)	4,040 (44.60%)	
Other	881 (4.07%)	578 (4.20%)		342 (3.78%)	362 (4.00%)	
Race			<0.001			0.008
White	18,057 (83.37%)	12,073 (87.78%)		7,906 (87.28%)	7,835 (86.50%)	
Black	1,988 (9.18%)	772 (5.61%)		577 (6.37%)	545 (6.02%)	
Other	1,613 (7.45%)	908 (6.60%)		575 (6.35%)	678 (7.49%)	
Involved lung lobes			<0.001			0.056
Lung segments	640 (2.96%)	33 (0.24%)		26 (0.29%)	33 (0.36%)	
Lobe	19,476 (89.93%)	13,386 (97.33%)		8,773 (96.85%)	8,724 (96.31%)	
Overlapping	172 (0.79%)	104 (0.76%)		57 (0.63%)	87 (0.96%)	
Lung	1,370 (6.33%)	230 (1.67%)		202 (2.23%)	214 (2.36%)	
Lung lobectomy			<0.001			<0.001
Left	9,153 (42.26%)	5,653 (41.10%)		3,955 (43.66%)	3,661 (40.42%)	
Right	12,182 (56.25%)	8,089 (58.82%)		5,094 (56.24%)	5,387 (59.47%)	
Bialateral	195 (0.90%)	6 (0.04%)		5 (0.06%)	6 (0.07%)	
Unknow	128 (0.59%)	5 (0.04%)		4 (0.04%)	4 (0.04%)	
Histologic Type			<0.001			<0.001
SQC	8,484 (39.17%)	3,802 (27.64%)		3,194 (35.26%)	2,967 (32.76%)	
ADC	10,120 (46.73%)	7,896 (57.41%)		4,736 (52.29%)	4,853 (53.58%)	
OC	3,054 (14.10%)	2,055 (14.94%)		1,128 (12.45%)	1,238 (13.67%)	
Grade			<0.001			<0.001
I–II	9,426 (43.52%)	9,107 (66.22%)		4,959 (54.75%)	4,737 (52.30%)	
III–IV	12,232 (56.48%)	4,646 (33.78%)		4,099 (45.25%)	4,321 (47.70%)	
AJCC-T			<0.001			0.527
T1–2	11,648 (53.78%)	11,595 (84.31%)		7,128 (78.69%)	7,093 (78.31%)	
T3–4	10,010 (46.22%)	2,158 (15.69%)		1,930 (21.31%)	1,965 (21.69%)	
AJCC-N			<0.001			0.022
N0	10,048 (46.39%)	11,320 (82.31%)		6,763 (74.66%)	6,628 (73.17%)	
N1–3	11,610 (53.61%)	2,433 (17.69%)		2,295 (25.34%)	2,430 (26.83%)	
AJCC-M			<0.001			0.948
M0	12,418 (57.34%)	13,251 (96.35%)		8,558 (94.48%)	8,556 (94.46%)	
M1	9,240 (42.66%)	502 (3.65%)		500 (5.52%)	502 (5.54%)	

### Analysis of OS survival in the modelling and validation groups of elderly patients with NSCLC

**Figure 1:**
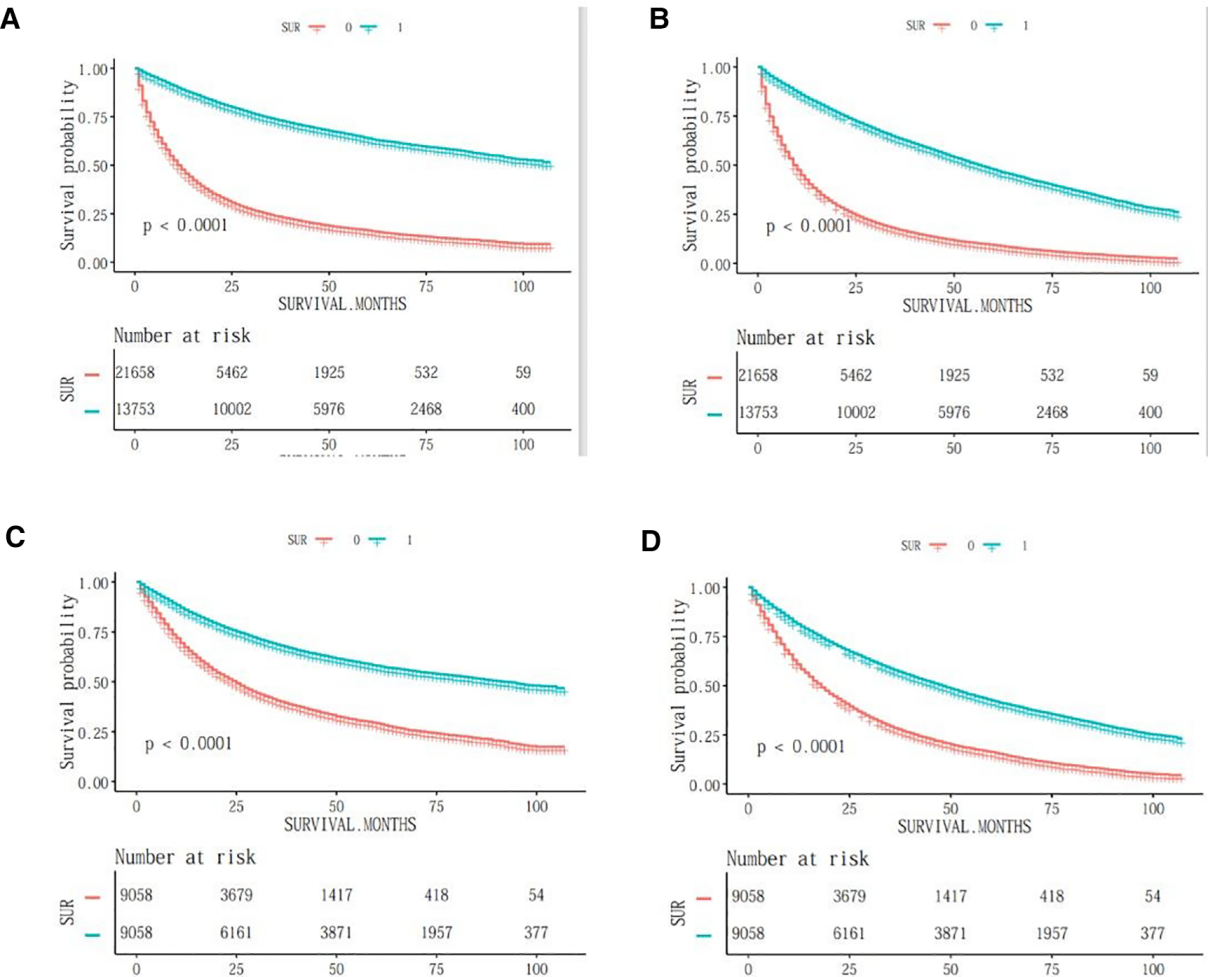
OS survival curves before and after PSM in elderly patients with NSCLC. (**A**) css-tumour-specific mortality before PSM; (**B**) os-tumour overall survival before PSM; (**C**) css-tumour-specific mortality after PSM; (**D**) os-tumour overall survival after PSM.

Kaplan-Meier plots visualise the differences in survival time after lobectomy in older patients with NSCLC. Overall, before and after propensity score matching (PSM), OS was shorter in the NSCLC high-risk mortality group than in the NSCLC non-high-risk group (*P* < 0.001). When comparing survival data between the two groups, survival rates were higher in the NSCLC non-high-risk group than in the NSCLC high-risk death group before and after matching, with survival rates of 73%, 43% and 18% at 25, 50 and 75 months in the NSCLC non-high-risk group and 25%, 8% and 2% at 25, 50 and 75 months in the NSCLC high-risk death group, respectively. Post-matching survival rates at 25, 50 and 75 months were 68%, 22% and 4% for the NSCLC non-high-risk group and 41%, 16% and 5% for the NSCLC high-risk mortality group, respectively. As shown in Figure 1.

### Screening for independent risk factors for survival in older patients with CSCLC

In the modeled group, the results of the one-way logistic regression analysis showed that the risk factors affecting the survival of elderly patients with confirmed lobectomy NSCLC were Sex, Age, Race, Histologic Type, and Grade (T, N, M), and the differences were all statistically significant at *P* < 0.05. These five risk factors were included again in the further multifactorial logistic regression analysis, and the results proved that these five risk factors were independent risk factors for survival in elderly patients with confirmed lobectomy NSCLC (Table 2).

**Table 2:** Univariable and multivariable logistic regression analysis in development group.

Variable	Statistics	Univariable		Multivariable	
		HR (95% CI)	*P*-value	HR (95% CI)	*P*-value
Sex					
Female	5,325 (51.86%)	ref		ref	
Male	4,944 (48.14%)	1.50 (1.19, 1.88)	0.0005	1.37 (1.08, 1.74)	0.0086
Age	79.28 ± 3.55	1.04 (1.00, 1.07)	0.0237	1.04 (1.01, 1.08)	0.0091
Marital status					
Married	5,472 (53.29%)	ref			
Signal	4,377 (42.62%)	0.86 (0.68, 1.08)	0.1935		
Other	420 (4.09%)	0.50 (0.23, 1.07)	0.073		
Race					
White	9,031 (87.94%)	ref		ref	
Black	563 (5.48%)	0.38 (0.18, 0.81)	0.0118	0.36 (0.17, 0.78)	0.0094
Other	675 (6.57%)	0.64 (0.37, 1.10)	0.1047	0.71 (0.41, 1.23)	0.2259
Involved lung lobes					
Lung segments	21 (0.20%)	ref			
Lobe	9,989 (97.27%)	0.28 (0.06, 1.20)	0.0861		
Overlapping	79 (0.77%)	1.38 (0.28, 6.83)	0.6954		
Lung. Nos	180 (1.75%)	0.86 (0.18, 4.07)	0.8529		
Lung lobectomy					
Left	4,216 (41.06%)	ref			
Right	6,044 (58.86%)	1.11 (0.88, 1.40)	0.3869		
Bialateral	6 (0.06%)	6.83 (0.79, 58.88)	0.0806		
Unknow	3 (0.03%)	0.00 (0.00, inf.)	0.9741		
Histologic type					
SQC	2,833 (27.59%)	ref		ref	
ADC	5,874 (57.20%)	0.62 (0.48, 0.79)	0.0001	0.66 (0.51, 0.86)	0.002
OC	1,562 (15.21%)	0.77 (0.55, 1.09)	0.1374	0.70 (0.49, 1.00)	0.0473
Grade					
I–II	6,774 (65.97%)	ref		ref	
III–IV	3,495 (34.03%)	2.10 (1.68, 2.64)	<0.0001	1.54 (1.21, 1.96)	0.0004
AJCC-T					
T1–2	8,659 (84.32%)	ref		ref	
T3–4	1,610 (15.68%)	2.78 (2.18, 3.54)	<0.0001	1.83 (1.41, 2.39)	<0.0001
AJCC-N					
N0	8,463 (82.41%)	ref		ref	
N1–3	1,806 (17.59%)	2.26 (1.77, 2.88)	<0.0001	1.71 (1.32, 2.22	<0.0001
AJCC-M					
M0	9,892 (96.33%)	ref		ref	
M1	377 (3.67%)	8.36 (6.23, 11.22)	<0.0001	6.00 (4.36, 8.25)	<0.0001
Preoperative RT					
Yes	71 (0.69%)	ref			
No	10,198 (99.31%)	1.08 (0.26, 4.42)	0.9169		

### Development of a line graph prediction model for elderly patients with NSCLC

**Figure 2:**
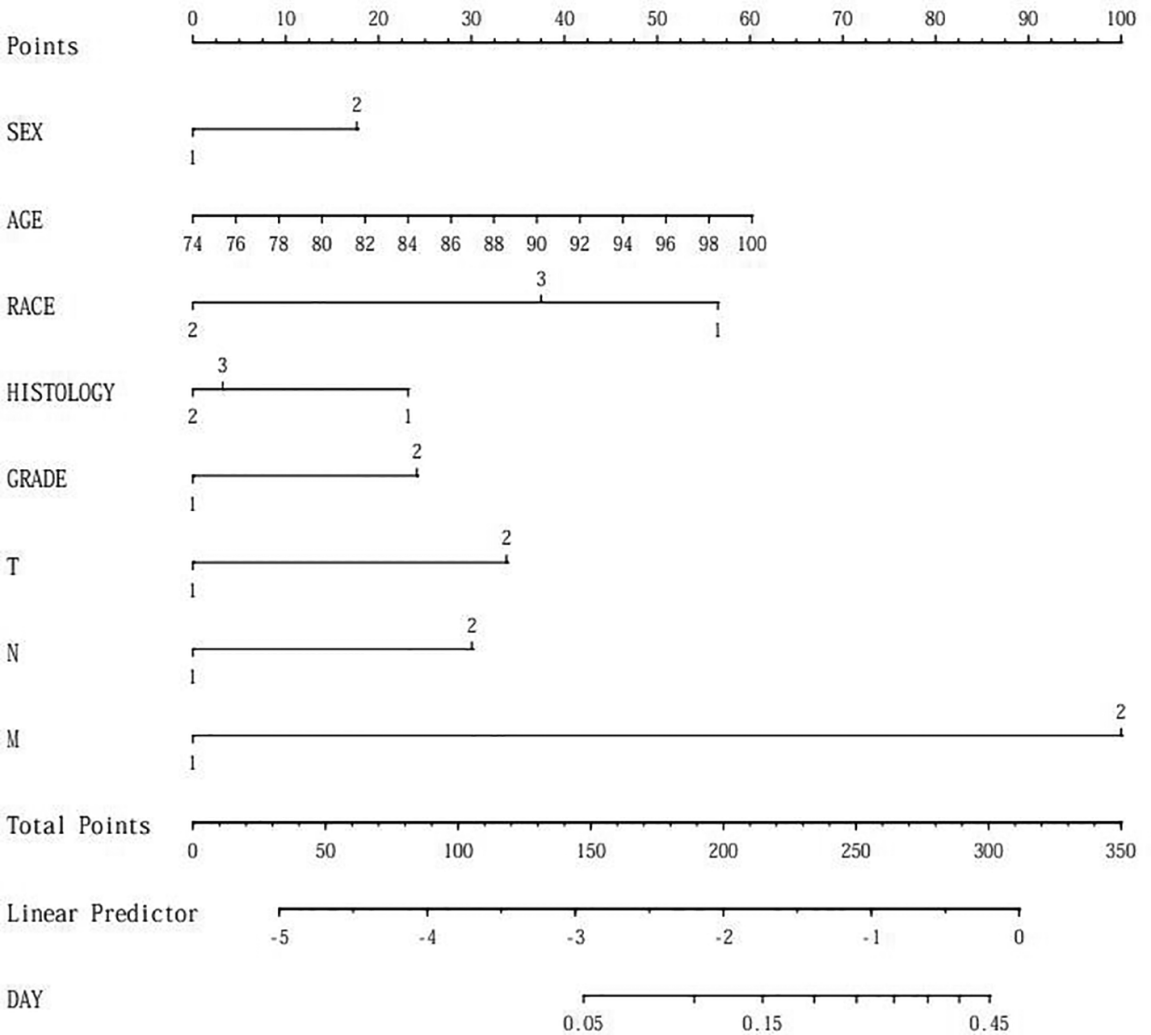
Predicted survival line graph for elderly patients undergoing lobectomy for NSCLC.

Based on the results of multi-factor logistic regression analysis, the survival of elderly patients diagnosed with lobectomy NSCLC was visualized and analyzed by including 5 factors affecting survival prognosis, sex, age, race, histology and grade, and a Nomogram prediction model was constructed based on the above 5 risk factors, as shown in Figure 2.

### Validation of the column line graph prediction model for elderly patients with NSCLC

**Figure 3:**
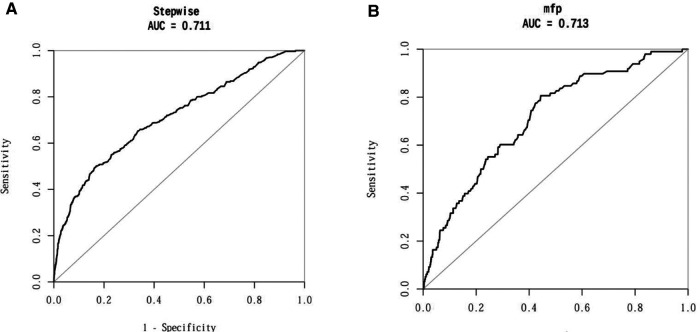
ROC curves of the prediction model for the column line graph of elderly patients with NSCLC. (**A**) AUC of the modelling group; (**B**) AUC of the validation group.

**Figure 4:**
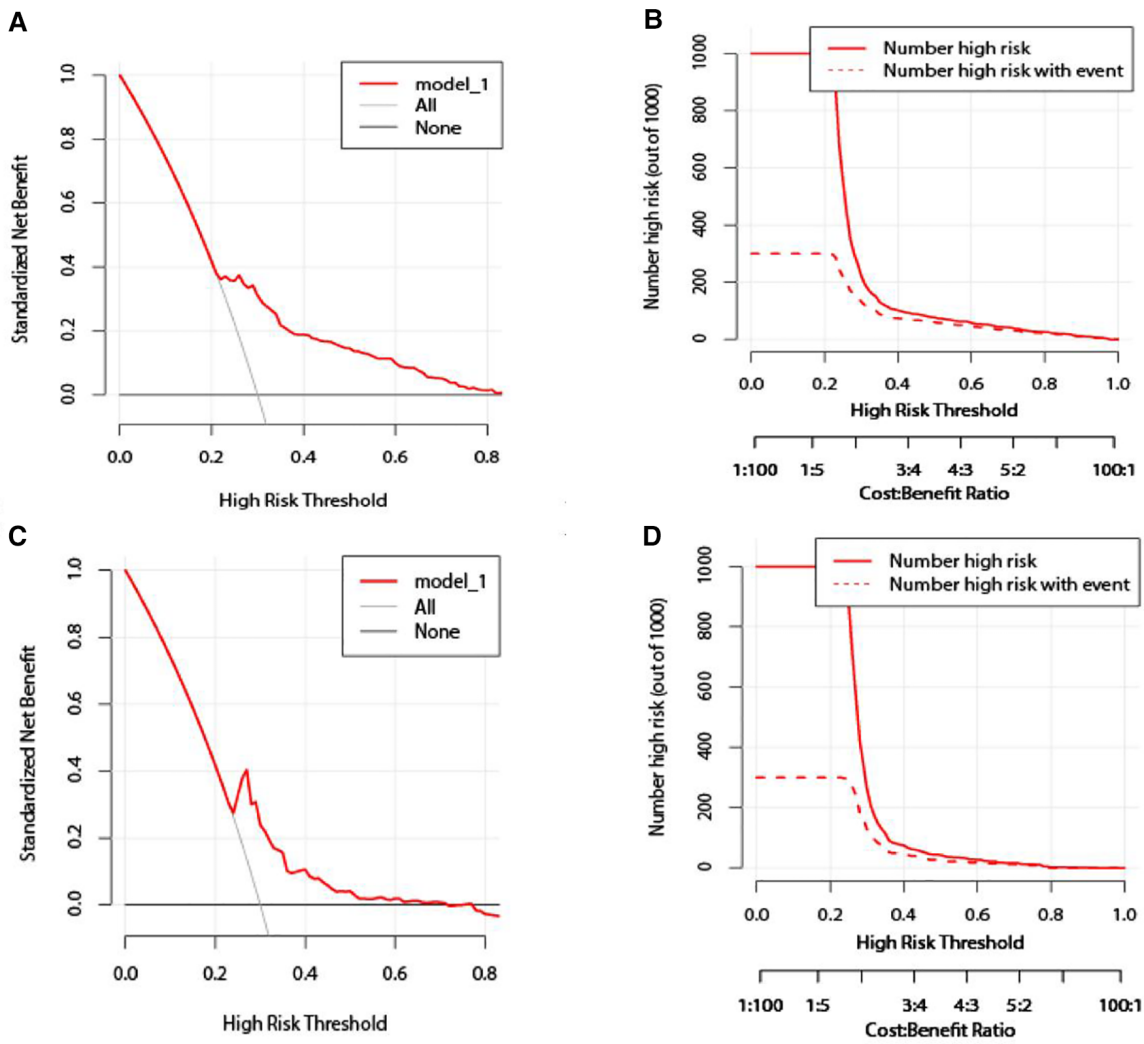
Validation of the column line graph prediction model. (**A,C**) Modeling group/validation group DCA decision curve; (**B,D**) modeling group/validation group clinical impact curve.

The Nomogram prediction model was validated by applying ROC curves, and the results showed that the area under the ROC curve for the modelling group prediction model was 0.711 AUC, and the area under the ROC curve for the validation group prediction model was 0.713 AUC, indicating that the Nomogram model has good prediction accuracy; as shown in Figures 3A,B. DCA decision curves and clinical impact curves are method to assess the value of the prediction model for clinical application. When the threshold probability of the DCA decision curve was shown to be between 1% and 90%, it indicated that the column line graph prediction model developed in this study had a high net clinical benefit, as shown in Figure 4.

## Discussion

Non-small cell lung cancer (NSCLC), is the leading cause of cancer deaths worldwide (10–12). Surgery is currently the main treatment for non-small cell lung cancer (NSCLC), with anatomical lobectomy being the main surgical treatment modality for NSCLC (13–15). With the development of lung cancer epidemiology and an ageing population, elderly patients with non-small cell lung cancer (NSCLC) have become an important patient group in lung cancer surgery. In this study, we analyzed the risk factors for postoperative mortality and the incidence of postoperative mortality in a special population of elderly patients undergoing lobectomy for NSCLC, and developed a practical prediction model to predict survival after lobectomy in elderly patients with NSCLC. The results of the study showed that the model has good predictive performance and has good clinical utility, providing guidance and decision making for the clinical survival of elderly patients undergoing lobectomy for NSCLC.

A prediction model (16) for NSCLC after radiotherapy has been proposed and predicts patient survival at 6 months, 1 year, 2 years and 3 years, but the model is only predictive of survival for patients treated with radiation for NSCLC. In the latest study on columnar graph prediction models for NSCLC, Chen (17) constructed a columnar graph model to predict prognosis of lymph node metastasis in NSCLC patients by four factors: age, SIRI, PNI and CEA. Zhang et al. (18) developed a line graph model to predict the development of advanced non-small cell lung cancer by combining clinical and molecular features. Xiaoping (19) developed a personalised line graph model based on CT-based sarcopenic to predict survival of patients with non-small cell lung cancer after receiving chemotherapy, which has some similarities to the present study. This study explored postoperative prognosis and survival model construction in NSCLC patients, but its study population was broad, none of which had predictive model construction regarding survival within 90 days after lobectomy in older (age ≥75 years) NSCLC patients.

As people continue to age, changes in the physiology and pathology of the elderly patient's organism are influenced by a number of factors, particularly physiological changes in the cardiovascular and respiratory systems (20, 21). This is mainly reflected in the reduced catecholamine response to stress, significantly reduced cardiac output, reduced lung tissue compliance, reduced expiratory effort and reduced pulmonary ventilation-perfusion capacity in elderly patients (22–24). In elderly patients with a long history of smoking, their vulnerability to hypoxaemia and hypercapnia presents a significant challenge for the surgical management of elderly patients with NSCLC (25–27). At the same time, the fact that patients older than 75 years of age often have many other underlying conditions (e.g., diabetes, hypertension, chronic bronchitis and emphysema) increases the risk of surgery and seriously affects the post-operative recovery of elderly patients.

We found that age, race, gender, degree of differentiation, histological type, and grade (T, N stage) were independent factors for OS in studies related to lung cancer, and these factors were consistent with findings on risk factors for non-small cell lung cancer (28–30). However, many previous studies have not clarified the multi-prognostic analysis of survival at 90 days postoperatively in elderly patients undergoing lobectomy for NSCLC (31–33). In this study, as many variables related to postoperative survival information for elderly patients undergoing lobectomy for NSCLC as possible will be included in the clinical prediction model to improve its accuracy. In this study, age and tumour infiltration grade were found to have a considerable impact on the survival of NSCLC patients. Guo et al. (34) showed that plasma-related pulmonary artery embolism levels in NSCLC patients increased significantly with increasing age, and that the prognostic hypercoagulable status of NSCLC patients was related to patients’ age, tumour infiltration grade and metastasis, and their postoperative treatment needed to take these factors into account. In addition, gender and ethnicity are important prognostic factors for patients with NSCLC, and gender is a strong prognostic factor when assessing the survival of patients with non-small cell lung cancer (35–37). Prognostic studies related to NSCLC (38–42) have also shown that the histologic subtype of NSCLC is associated with the risk of postoperative death in NSCLC patients, and that the overall postoperative survival OS of patients with different non-small cell lung cancer (NSCLC) histologic subtypes varies. Therefore, we identified Sex, Age, Race, Histologic Type, and Grade (T, N, M) as variables used in the column line graph, and the column line graph model constructed by these variables is of great importance in predicting the survival rate of elderly patients after lobectomy for NSCLC and guiding their treatment.

In this study, we constructed a survival prediction line graph model for elderly patients after lobectomy for NSCLC, which was based on a large database population to predict OS in patients undergoing lobectomy for NSCLC. in the model testing and validation, we found that the area under the ROC curve AUC of the model was 0.711 and 0.713 in the testing and validation groups, respectively, which were both much greater than 0.5, indicating that the model was predictive of survival of elderly patients after lobectomy for NSCLC is predictive efficacy and the model has good predictive performance. The clinical utility of the model was assessed by DCA decision curves and clinical impact curves, and the results of the validation group showed that the column line graph prediction model developed in our study has a high net clinical benefit and can be used as a tool for clinical decision making. Physicians and patients can use the column line graph model prediction scoring system to understand the individualised survival expectations of elderly patients after lobectomy for NSCLC, and based on the results of the column line graph prediction, effective interventions can be made to improve the survival of elderly patients after lobectomy for NSCLC with low survival rates.

This study was enrolled in the National Cancer Institute's SEER database, and although the sample size was large, there were some limitations. (1) This study is a retrospective study with a low level of evidence compared to prospective cohort studies. (2) In order to eliminate the influence of confounding factors on the study results, the data were processed using propensity score matching (PSM), but there was still an unavoidable selective bias in the two data sets. (3) This study is a single-centre study, with both the test and validation sets from the same database, and lacks external datasets to validate the prediction model of this study. Our next research direction is to conduct a multicentre, large sample size prospective study of elderly patients after lobectomy for NSCLC. (4) The SEER database does not provide more information on elderly patients undergoing lobectomy for NSCLC, such as cause of death, surgical approach, and postoperative adjuvant treatment options, which may also be important factors influencing the information on postoperative survival of elderly patients with NSCLC. Despite some limitations, the Nomogram was constructed based on a large population and provides a practical and effective clinical tool for predicting survival in elderly patients undergoing lobectomy for NSCLC.

## Conclusion

In this study, sex, age, race, histology and grade were important factors influencing postoperative survival in elderly patients after lobectomy for NSCLC. For elderly patients after lobectomy for NSCLC, timely and effective postoperative survival assessment and enhanced clinical supervision are beneficial to improve the survival rate of elderly patients after lobectomy for NSCLC.

## Data Availability

The original contributions presented in the study are included in the article/Supplementary Material, further inquiries can be directed to the corresponding author.
